# Supplementary material for: HIVprotI: an integrated web based platform for prediction and design of HIV proteins inhibitors
Source: J Cheminform. 2018 Mar 9;10:12. doi: 10.1186/s13321-018-0266-y (PMC5845081; doi:10.1186/s13321-018-0266-y)
Supplement: Supplementary file 2 — Additional file 2. Source code of HIVProtI web server. [file 13321_2018_266_MOESM2_ESM.zip › HIVprotI_Source-code/hivprotI/weka-3-6-6/doc/weka/classifiers/evaluation/ThresholdCurve.html]

ThresholdCurve


---


|  |  |  |
| --- | --- | --- |
| **Overview** \| **Package** \| **Class** \| **Tree** \| **Deprecated** \| **Index** \| **Help** \| **Weka's home** | |  |
| **PREV CLASS**   **NEXT CLASS** | **FRAMES**    **NO FRAMES**     **All Classes** |  |
| SUMMARY: NESTED \| FIELD \| CONSTR \| METHOD | DETAIL: FIELD \| CONSTR \| METHOD |  |


---


## weka.classifiers.evaluation Class ThresholdCurve

```
java.lang.Object
  weka.classifiers.evaluation.ThresholdCurve
```

**All Implemented Interfaces:**: RevisionHandler

---

``` public class ThresholdCurve extends java.lang.Object implements RevisionHandler ```

Generates points illustrating prediction tradeoffs that can be obtained
by varying the threshold value between classes. For example, the typical
threshold value of 0.5 means the predicted probability of "positive" must be
higher than 0.5 for the instance to be predicted as "positive". The
resulting dataset can be used to visualize precision/recall tradeoff, or
for ROC curve analysis (true positive rate vs false positive rate).
Weka just varies the threshold on the class probability estimates in each
case. The Mann Whitney statistic is used to calculate the AUC.

**Version:**
:   $Revision: 5787 $

**Author:**
:   Len Trigg (len@reeltwo.com)

---

| **Field Summary** | |
| --- | --- |
| `static java.lang.String` | `FALLOUT_NAME`             attribute name: Fallout |
| `static java.lang.String` | `FALSE_NEG_NAME`             attribute name: False Negatives |
| `static java.lang.String` | `FALSE_POS_NAME`             attribute name: False Positives |
| `static java.lang.String` | `FMEASURE_NAME`             attribute name: FMeasure |
| `static java.lang.String` | `FP_RATE_NAME`             attribute name: False Positive Rate" |
| `static java.lang.String` | `LIFT_NAME`             attribute name: Lift |
| `static java.lang.String` | `PRECISION_NAME`             attribute name: Precision |
| `static java.lang.String` | `RECALL_NAME`             attribute name: Recall |
| `static java.lang.String` | `RELATION_NAME`             The name of the relation used in threshold curve datasets |
| `static java.lang.String` | `SAMPLE_SIZE_NAME`             attribute name: Sample Size |
| `static java.lang.String` | `THRESHOLD_NAME`             attribute name: Threshold |
| `static java.lang.String` | `TP_RATE_NAME`             attribute name: True Positive Rate |
| `static java.lang.String` | `TRUE_NEG_NAME`             attribute name: True Negatives |
| `static java.lang.String` | `TRUE_POS_NAME`             attribute name: True Positives |


**Constructor Summary**

`ThresholdCurve()`


| **Method Summary** | |
| --- | --- |
| `Instances` | `getCurve(FastVector predictions)`             Calculates the performance stats for the default class and return results as a set of Instances. |
| `Instances` | `getCurve(FastVector predictions, int classIndex)`             Calculates the performance stats for the desired class and return results as a set of Instances. |
| `static double` | `getNPointPrecision(Instances tcurve, int n)`             Calculates the n point precision result, which is the precision averaged over n evenly spaced (w.r.t recall) samples of the curve. |
| `java.lang.String` | `getRevision()`             Returns the revision string. |
| `static double` | `getROCArea(Instances tcurve)`             Calculates the area under the ROC curve as the Wilcoxon-Mann-Whitney statistic. |
| `static int` | `getThresholdInstance(Instances tcurve, double threshold)`             Gets the index of the instance with the closest threshold value to the desired target |
| `static void` | `main(java.lang.String[] args)`             Tests the ThresholdCurve generation from the command line. |

**Methods inherited from class java.lang.Object**

`equals, getClass, hashCode, notify, notifyAll, toString, wait, wait, wait`

**Field Detail**

### RELATION\_NAME

```
public static final java.lang.String RELATION_NAME
```

:   The name of the relation used in threshold curve datasets

    **See Also:**: Constant Field Values

---


### TRUE\_POS\_NAME

```
public static final java.lang.String TRUE_POS_NAME
```

:   attribute name: True Positives

    **See Also:**: Constant Field Values

---


### FALSE\_NEG\_NAME

```
public static final java.lang.String FALSE_NEG_NAME
```

:   attribute name: False Negatives

    **See Also:**: Constant Field Values

---


### FALSE\_POS\_NAME

```
public static final java.lang.String FALSE_POS_NAME
```

:   attribute name: False Positives

    **See Also:**: Constant Field Values

---


### TRUE\_NEG\_NAME

```
public static final java.lang.String TRUE_NEG_NAME
```

:   attribute name: True Negatives

    **See Also:**: Constant Field Values

---


### FP\_RATE\_NAME

```
public static final java.lang.String FP_RATE_NAME
```

:   attribute name: False Positive Rate"

    **See Also:**: Constant Field Values

---


### TP\_RATE\_NAME

```
public static final java.lang.String TP_RATE_NAME
```

:   attribute name: True Positive Rate

    **See Also:**: Constant Field Values

---


### PRECISION\_NAME

```
public static final java.lang.String PRECISION_NAME
```

:   attribute name: Precision

    **See Also:**: Constant Field Values

---


### RECALL\_NAME

```
public static final java.lang.String RECALL_NAME
```

:   attribute name: Recall

    **See Also:**: Constant Field Values

---


### FALLOUT\_NAME

```
public static final java.lang.String FALLOUT_NAME
```

:   attribute name: Fallout

    **See Also:**: Constant Field Values

---


### FMEASURE\_NAME

```
public static final java.lang.String FMEASURE_NAME
```

:   attribute name: FMeasure

    **See Also:**: Constant Field Values

---


### SAMPLE\_SIZE\_NAME

```
public static final java.lang.String SAMPLE_SIZE_NAME
```

:   attribute name: Sample Size

    **See Also:**: Constant Field Values

---


### LIFT\_NAME

```
public static final java.lang.String LIFT_NAME
```

:   attribute name: Lift

    **See Also:**: Constant Field Values

---


### THRESHOLD\_NAME

```
public static final java.lang.String THRESHOLD_NAME
```

:   attribute name: Threshold

    **See Also:**: Constant Field Values


**Constructor Detail**

### ThresholdCurve

```
public ThresholdCurve()
```


**Method Detail**

### getCurve

```
public Instances getCurve(FastVector predictions)
```

:   Calculates the performance stats for the default class and return
    results as a set of Instances. The
    structure of these Instances is as follows:

    - **True Positives** - **False Negatives**- **False Positives**- **True Negatives**- **False Positive Rate**- **True Positive Rate**- **Precision**- **Recall**- **Fallout**- **Threshold** contains the probability threshold that gives
                        rise to the previous performance values.

    For the definitions of these measures, see TwoClassStats

    :   **Parameters:**: `predictions` - the predictions to base the curve on **Returns:**: datapoints as a set of instances, null if no predictions have been made. **See Also:**: `TwoClassStats`

---


### getCurve

```
public Instances getCurve(FastVector predictions,
                          int classIndex)
```

:   Calculates the performance stats for the desired class and return
    results as a set of Instances.

    :   **Parameters:**: `predictions` - the predictions to base the curve on: `classIndex` - index of the class of interest. **Returns:**: datapoints as a set of instances.

---


### getNPointPrecision

```
public static double getNPointPrecision(Instances tcurve,
                                        int n)
```

:   Calculates the n point precision result, which is the precision averaged
    over n evenly spaced (w.r.t recall) samples of the curve.

    :   **Parameters:**: `tcurve` - a previously extracted threshold curve Instances.: `n` - the number of points to average over. **Returns:**: the n-point precision.

---


### getROCArea

```
public static double getROCArea(Instances tcurve)
```

:   Calculates the area under the ROC curve as the Wilcoxon-Mann-Whitney statistic.

    :   **Parameters:**: `tcurve` - a previously extracted threshold curve Instances. **Returns:**: the ROC area, or Double.NaN if you don't pass in a ThresholdCurve generated Instances.

---


### getThresholdInstance

```
public static int getThresholdInstance(Instances tcurve,
                                       double threshold)
```

:   Gets the index of the instance with the closest threshold value to the
    desired target

    :   **Parameters:**: `tcurve` - a set of instances that have been generated by this class: `threshold` - the target threshold **Returns:**: the index of the instance that has threshold closest to the target, or -1 if this could not be found (i.e. no data, or bad threshold target)

---


### getRevision

```
public java.lang.String getRevision()
```

:   Returns the revision string.

    :   **Specified by:**: `getRevision` in interface `RevisionHandler`
    :   **Returns:**: the revision

---


### main

```
public static void main(java.lang.String[] args)
```

:   Tests the ThresholdCurve generation from the command line.
    The classifier is currently hardcoded. Pipe in an arff file.

    :   **Parameters:**: `args` - currently ignored


---


|  |  |  |
| --- | --- | --- |
| **Overview** \| **Package** \| **Class** \| **Tree** \| **Deprecated** \| **Index** \| **Help** \| **Weka's home** | |  |
| **PREV CLASS**   **NEXT CLASS** | **FRAMES**    **NO FRAMES**     **All Classes** |  |
| SUMMARY: NESTED \| FIELD \| CONSTR \| METHOD | DETAIL: FIELD \| CONSTR \| METHOD |  |


---
